# The Contribution of the Triage Nurse in the Optimisation of Door-to-Computed-Tomography Time in Stroke

**DOI:** 10.3390/nursrep14030131

**Published:** 2024-07-17

**Authors:** Raquel Antunes, Cristina Costeira, Joana Pereira Sousa, Cátia Santos

**Affiliations:** 1 Local Health Unit of Castelo Branco, 6000-085 Castelo Branco, Portugal; raquel.antunes@ulscb.min-saude.pt; 2 School of Health Sciences, Polytechnic of Leiria, Center for Innovative Care and Health Technology—ciTechCare, 2411-901 Leiria, Portugal; cristina.costeira@ipleiria.pt (C.C.); joana.sousa@ipleiria.pt (J.P.S.); 3 Health Sciences Research Unit: Nursing (UICISA: E), Nursing School of Coimbra (ESEnfC), 3004-011 Coimbra, Portugal

**Keywords:** stroke, nurse, triage, emergency

## Abstract

A stroke is a time-sensitive emergency, so diagnosing and treating the victim promptly is extremely important. Therefore, the purpose of this study was to identify the influence of the Stroke Code Protocol’s activation on the door-to-computed-tomography (door-to-CT) time and determine whether factors such as previous Modified Rankin Scale (mRS), age, and gender influence its activation. A retrospective study was conducted in a Medical-Surgical Emergency Department in the centre of Portugal from 1 January 2021 to 31 December 2022. The sample was selected according to the diagnosis assigned at the time of clinical discharge from the Emergency Department and the Stroke Code Protocol activation criteria. It was observed that 113 (50%) suspected stroke victims who met the activation criteria for the Stroke Code Protocol did not have the protocol activated, which had a highly significant influence (*p* < 0.001) on door-to-CT time. It was determined that activation at triage has an average door-to-CT time of 35 ± 18 min, post-triage activation has an average door-to-CT time of 38 ± 26 min, and non-activation has an average door-to-CT time of 1 h 04 ± 45 min. The need to implement an institutional protocol for activating the Stroke Code Protocol and provide specialised training for the multidisciplinary team is reiterated.

## 1. Introduction

Over the past two decades, stroke incidence has decreased in Europe, and the likelihood of recovery for victims has significantly improved, making Europe a global pioneer in developing and enhancing stroke care. Despite this progress, studies predict an increase in stroke incidence and prevalence due to rising life expectancy in the coming decades [1,2,3,4,5]. Stroke remains the second leading cause of death worldwide, with approximately 12.2 million new cases annually. Furthermore, one in four people over the age of 25 are expected to suffer a stroke at some point in their lives [3]. This alarming statistic underscores the imperative for continued research and development in stroke management and preventive measures. In Portugal, stroke is the leading cause of death among circulatory system diseases, with around 10,000 deaths in 2021 [6]. Prompt diagnosis and treatment are critical as every minute of large cerebral blood vessel occlusion results in the loss of an estimated 1.9 million neurons, equivalent to 3.6 years of natural ageing each hour [7]. The ‘golden hour’ following a stroke is crucial for successful outcomes, highlighting the need for efficient treatment protocols. The Stoke Alliance for Europe (SAFE) aims to provide equal access to stroke prevention, diagnosis, treatment, rehabilitation, and long-term support throughout Europe. By 2030, SAFE’s goals include that at least 90% of stroke victims receive care in stroke units during the acute phase, with door-to-needle times of less than 120 min and symptom onset to thrombectomy times of less than 200 min [8].

In Portugal, it is recognised that Emergency Departments (EDs) need to have priority triage systems in place. These systems help distinguish the degree of clinical priority of each patient, ensuring that they are seen by the most appropriate team as soon as possible [9,10]. The triage post is managed by a nurse who has undergone specific training in the Priority Triage System. As per the recommendation of the Order of Nurses, they should specialise in Medical-Surgical Nursing in Critical Care Nursing [11]. This specialised training enables triage nurses to make swift, accurate decisions, which can be the difference between life and death or disability and recovery for stroke patients, which ensures quality and safe healthcare based on international best practises. The nurse’s level of qualification and skillset is of utmost importance, as it is a fundamental prerequisite for quick and accurate victim triage. The triage nurse plays a crucial role in determining and prioritising the person’s needs when faced with a life-threatening situation that requires immediate observation [12].

To improve the approach to individuals suspected of having a stroke, the Directorate-General of Health of Portugal [DGS] has issued standard no. 015/2017 titled “*Via Verde do Acidente Vascular Cerebral no Adulto*” (VVAVC), translated as “Greenway for Stroke in Adults”. This standard represents a strategic plan for the approach to, referral of, and treatment of suspected stroke victims on time and falls under the international umbrella of the Stroke Code Protocol. It includes pre-hospital, in-hospital, and inter-hospital phases and is designed to improve the quality of care provided to stroke victims. The VVAVC must be activated when a victim presents one of the signs/symptoms of the Cincinnati Scale. Additionally, the standard defines the quality-of-care indicators that must be continuously monitored, including the door-to-computed-tomography (door-to-CT) time [13].

The stroke care pathway, from early detection to rehabilitation, underscores the need for a comprehensive approach to address this complex health issue. VVAVC activation aims to promote the health and well-being of suspected stroke victims using a person-centred approach. It emphasises the provision of high-quality nursing care based on scientific evidence and research in close collaboration within the healthcare team. The triage nurse plays a crucial role as the first point of contact for suspected stroke victims arriving at the ED. Therefore, the main aim of this study was to determine how the moment of VVAVC (Greenway for Stroke) activation by the triage nurse affects the door-to-CT time.

Additionally, accordingly to the fact that older and disabled patients are typically not considered for intravenous thrombolysis (IVT) treatment due to concerns about poor outcomes, recent stroke guidelines and studies suggest that IVT may be reasonable for patients over 80 years old and those with previous Modified Rankin Scale (mRS) score of ≥2, with some studies showing favourable outcomes and significant neurological improvement compared to untreated patients [14,15,16,17,18,19,20,21]. This study also aims to investigate whether factors such as previous mRS score, age, and gender influence the decision to activate the VVAVC.

## 2. Materials and Methods

A retrospective study was conducted in the Medical-Surgical Emergency Department of a Local Health Unit (ULS) in the Centre region of Portugal, Group B, covering the period from 1 January 2021 to 31 December 2022.

The study population included individuals over 18 who, at the time of discharge from the ED, had a diagnosis between codes I60 and I69 (cerebrovascular diseases) according to the ICD-10 (*International Classification of Diseases 10th Revision*).

To categorise the study, we identified independent and dependent variables. The independent variable was the door-to-CT time, while the dependent variables fell into three groups: sociodemographic factors (age and gender), clinical factors before admission (including previous mRS and origin), and clinical factors after admission (such as shift and the time of VVAVC activation). We grouped the age variable into quartiles and stratified the remaining variables based on clinical severity at triage.

### 2.1. Sample

The criteria for selecting the sample were as follows: The presence of a sign or symptom from the Cincinnati Scale at triage and the evolution of the symptomatology up to 12 h. This window was selected based on the acute phase of stroke, where intervention is most critical. The “time to symptom onset” criterion was based on the availability of only computed tomography (CT) as an imaging test at the institution, along with the fibrinolysis and thrombectomy timings referred to in 2021 [14] and 2019 [15] European Stroke Association (ESO) guidelines, respectively.

### 2.2. Data Collection

The data for this study were collected from the patient’s medical and nursing records and provided by the institution in an EXCEL^®^ file format (Microsoft Corporation, Redmond, WA, USA), with authorisation from its Ethics Committee. Compliance with the General Data Protection Regulation was ensured, and utmost care was taken to maintain the anonymity of the institution and the confidentiality of all personal data collected.

### 2.3. Statistical Analysis

Statistical data analysis was conducted using IBM SPSS^®^ software, version 29.0.1.0. A *p*-value of <0.05 was considered statistically significant. The analysis began with a descriptive sample examination based on sociodemographic and clinical data before and after admission to the ED by year. Normality tests were conducted to determine the suitability of parametric versus non-parametric tests for inferential analysis. Inferential analysis was then performed to identify statistically significant differences between the variables. Two questions were posed: Does the moment of activation of VVAVC have an impact on the door-to-CT time? Do sociodemographic variables (age and gender) and the clinical variable (previous mRS) have an impact on the activation of VVAVC?

## 3. Results

### 3.1. Descriptive Analysis

**Figure 1:**
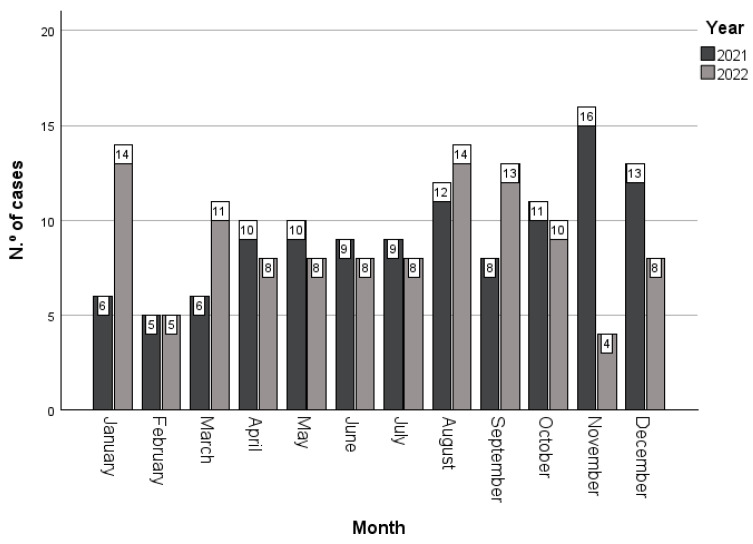
Characterisation of cases identified by month and year (2021–2022).

From a total population of 553 cases (100%), 273 (49.4%) occurred in 2021 and 280 (50.6%) in 2022. By applying the above criteria, a total sample of 226 cases (100%) was obtained: 115 (50.9%) in 2021 and 111 (49.1%) in 2022. Regarding the monthly cases per year, November 2021 recorded the highest number, with 16 (13.9%), while January and August 2022 stood out with 14 (12.6%) each. The total number of cases in August was the highest in both years, with 26 (11.5%). No significant differences existed between the months and years (X^2^ = 14.949; *p* = 0.185) (Figure 1).

#### 3.1.1. Sociodemographic Characterisation

The sample’s average age was 79.81 ± 11.96, with the youngest participant being 38 and the oldest being 103. It is worth noting that the average age in 2022 (80.32 ± 12 years) was higher than in 2021 (79.31 ± 11.96 years). Most cases were between 73 and 82 years of age (*n* = 61; 27%). No significant differences were found between age and year, a situation proven by the distribution of residual values and the chi-squared test (X^2^ = 2.774; *p* = 0.428) (Table 1).

Concerning the gender variable, there were more cases of male victims in both years, with 121 (53.5%) cases over the two years. The distribution was similar in both years, indicating no significant differences (X^2^ = 0.013; *p* = 0.909). The distribution of residual values in Table 1, with all values falling between −1.96 and 1.96, which is statistically not significant, further confirm this.

#### 3.1.2. Characterisation of Clinical Data (Pre-Admission)

Regarding the previous mRS score, before the ED episode, 159 patients (70.4%) were asymptomatic. This distribution was consistent across both years.

In terms of patient origin, “Outside” refers to patients who arrived at the hospital on their own, “Centre of Portuguese Urgent Patients Orientation (CODU)” refers to patients who called the emergency number and were transported to the hospital by ambulance, “Health Centre” refers to patients who initially sought care at a primary care facility and were then sent to the hospital. Most patients, 184 (81.4%), were referred by the CODU. This distribution was similar across both years (see Table 2).

#### 3.1.3. Characterisation of Clinical Data (Post-Admission)

Regarding the admission shift, the morning shift (8 am to 4 pm) shows the highest representation with 130 (57.5%) cases. This distribution remained consistent in both 2021 and 2022. The analysis found no significant differences between shift and year (X^2^ = 4.345; *p* = 0.114), supported by the distribution of residual values (Table 3).

Of all the cases considered for VVAVC activation, 113 (50%) were not activated. This distribution remained consistent in both years. Out of the 113 cases activated, 83 (36.7%) were activated at the time of triage, while the remaining 30 (13.3%) were activated after triage. However, we found statistically significant differences between the moment of activation and the year (X^2^ = 8.317; * *p* = 0.016), which is also evident from the distribution of residual values. In 2021, the frequency of activations at triage was lower than expected (residual = −2.8), while in 2022, the frequency of triage activations was higher than expected (residual = 2.8). A similar situation occurred with non-activations: in 2021, the frequency was higher than expected (residual = 2.5), while in 2022, the frequency was lower than expected (residual = −2.5). In both situations, the data are statistically significant (Table 3).

Based on the analysed data, it was found that the average time taken from admission to triage was 8 ± 5 min. The average door-to-CT time for the two years was 50 ± 37 min. In 2021, it was 52 ± 44 min, while in 2022, it was 47 ± 29 min. The average door-to-CT time showed a similar trend in both years, being lower when VVAVC was activated during triage and higher when it was not. However, it is worth noting that in 2022, the average door-to-CT time with activation at triage and post-triage increased compared to 2021. The average time from admission to activation of VVAVC was around 5 ± 16 min in 113 (50%) cases (Table 4).

### 3.2. Inferential Analysis

Question 1: Does the moment of VVAVC activation affect the door-to-CT time?

A Kruskal–Wallis test determined that the timing of VVAVC activation significantly impacted door-to-CT time. The results indicated a significant difference between triage activation, post-triage activation, and non-activation. Activation during triage resulted in an average door-to-CT time of 35 ± 18 min, while post-triage activation had an average door-to-CT time of 38 ± 26 min. These times were significantly lower compared to cases where the protocol was not activated, which had an average door-to-CT time of 1 h 04 ± 45 min (* *p* = 0.000) (Table 5).

Question 2: Do the sociodemographic variables (age and gender) and the clinical variable (previous mRS) influence VVAVC activation?

Regarding the influence of age on the activation of VVAVC, we observed that the 83–89 age group had a higher number of VVAVC activations at triage, while the under-72 age group had more post-triage activations. Non-activated cases were more prevalent in the 73–82 age group. In terms of gender, VVAVC activation at triage was higher in men, while post-triage activation was higher in women. Additionally, non-activation of the VVAVC was more common in men. Most cases had no previous symptoms (mRS = 0), regardless of VVAVC activation. However, when the previous mRS score was high, there was a tendency for lower activations at triage and post-triage and an increase in non-activations. In conclusion, the analysis revealed no significant effects of age, gender, or previous mRS score on the activation of the VVAVC protocol (Table 6).

## 4. Discussion

The study found that August had the highest number of cases in both years, with 26 (11.5%) cases. There were no significant differences between the month and year. As no studies on the incidence of strokes according to month were found in Portugal, we compared the data with the INE, which studies mortality from cerebrovascular diseases according to month. It showed that the highest number of deaths in 2020 occurred in January, March, and April [6]. A similar trend was observed in 2022, where January had the highest incidence of cases (n = 14;12.6%).

Regarding the age variable, the analysis showed that the age difference between the study years was not statistically significant (X^2^ = 2.774; *p* = 0.428). Therefore, no significant age differences were found between the participants in the two years.

In terms of gender variables, there was a similar distribution between male and female participants over both years, with more male victims—totalling 121 (53.5%) cases. No statistically significant differences were found (X^2^ = 0.013; *p* = 0.909). A similar study conducted by Barreira (2018) in the north of Portugal between 2010 and 2016 with a sample of 1200 people with cerebrovascular diseases found an average age of 77.4 ± 11.2 years, with more male cases (n = 658; 54.8%) [16].

In Portugal, due to the lack of recent studies on the subject, we analysed the age and gender variables of stroke victims and compared the obtained data with INE data from 2020. The data show that the number of deaths from cerebrovascular diseases increases with age, with a higher number of cases in the 85+ age group. Women have a higher overall mortality rate, but among those aged 65–79, the rate is higher among men [6].

Combined with the fact that the average age increased from 2021 to 2022, there was a considerable number of suspected stroke victims with a previous asymptomatic mRS (n = 159; 70.4%). This may be related to the increase from 8.3% to 16.6% of the Portuguese population aged over 65 with a health status classified as very good to good between 2004 and 2022, as well as the average life expectancy in the centre of the country standing at 81.34 years [6,17]. This could indicate a population with undetected or poorly managed risk factors, pointing towards the need for improved public health strategies in early risk factor identification and management [18].

Out of the total number of stroke cases, 184 (81.4%) were referred to the ED by CODU under the standard DGS set. However, this study could not obtain data on pre-hospital VVAVC activations, a limitation pointing to the need for a more integrated data collection system across different phases of stroke care in Portugal.

Regarding pre-hospital VVAVC activations in Portugal, Lavinha’s (2019) [19] study discovered that the number of pre-hospital VVAVC activations was only 6194 (12.97%). This was a tiny percentage compared to the total number of urgently admitted stroke hospitalisations, which was 49,047. He also mentioned a moderate correlation between the number of symptoms and the use of the pre-hospital VVAVC in municipalities with older populations, fewer university graduates, and higher illiteracy rates. The results showed a relationship between the difficulty of the population in identifying stroke symptoms and the poor use of the emergency system [19].

Our study analysed the use of in-hospital VVAVC based on the moment of its activation. We categorised activation into three types: activation during triage, post-triage activation, and non-activation. Out of 226 cases, VVAVC was activated 113 (50%) times. Among the activated cases, 83 (36.7%) activations occurred during triage, while 30 (13.3%) activations happened after. These figures are similar to those found in a previous study by Barreira (2018), where only 431 (35.9%) activations were identified in a sample of 1200 people [16].

The analysis of various times found that the average time from admission to triage was 8 ± 5 min, the average time from admission to the activation of VVAVC was 5 ± 16 min, and the average door-to-CT time was 50 ± 37 min. Optimising the time factor is crucial when dealing with suspected stroke victims. However, this study did not address the place of hospital admission for victims with pre-hospital activated VVAVC. Meanwhile, Lavinha (2019) [19] surveyed the entry points for stroke victims and found that most cases (42.1%) were transported to pre-triage, followed by the resuscitation room (34.1%), and only 10.6% were sent directly to the imaging service [19]. Madhok et al. (2019) found that referring patients directly from the ambulance to the imaging service significantly reduced the average door-to-CT time [20]. Therefore, it is vital to conduct future studies to determine the ideal entry point for suspected stroke victims in Portugal’s Emergency Departments, as well as create and implement intra-hospital circuits to improve the time factor.

The impact of VVAVC activations on door-to-CT times in our study echoes the findings from international studies that stress the importance of swift initial management in the ED. The American Stroke Association’s Target: this stroke initiative is a benchmark for evaluating the efficacy of similar protocols worldwide, emphasising the value of quick response in improving stroke outcomes [22].

In response to the first question about whether the moment of activation of VVAVC affects the door-to-CT time, an impact was discovered in 113 (50%) cases where the VVAVC was not activated in suspected stroke patients who met the activation criteria. This non-activation had a highly significant impact (*p* < 0.001) on the door-to-CT time. It was determined that activation during triage resulted in an average door-to-CT time of 35 ± 18 min, activation after triage led to an average door-to-CT time of 38 ± 26 min, and non-activation resulted in an average door-to-CT time of 1 h 04 ± 45 min. Further research is needed to explore the barriers to protocol activation and devise strategies to overcome them, as timely imaging is crucial for effectively managing stroke victims.

It has been observed that the number of VVAVC activations during triage increased from 32 (27.8%) in 2021 to 51 (45.9%) in 2022. At the same time, the number of non-activations decreased from 67 (58.3%) to 46 (41.4%). However, it was also noticed that the average door-to-CT time on an activated VVAVC, both at triage and post-triage, increased from 2021 to 2022. The study did not establish any causal relationship between these conditions. Therefore, further studies are required to understand these changes better.

Based on the data above and the importance of quality in healthcare, acting fast in case of a stroke is crucial. The victim’s health outcomes depend on how quickly they are approached, diagnosed, and treated. To address this issue, the American Stroke Association (ASA) and the American Heart Association (AHA) have created various initiatives since 2010. Their goal is to establish increasingly shorter time targets. The most recent one is Target: Stroke Phase III, which recommends a door-to-CT time of less than or equal to 15 min [23].

As such, studies have implemented strategies and protocols to optimise door-to-CT time to reduce the time factor. Kazi et al. (2022) conducted a study where they trained stroke triage nurses, which helped them reduce the average door-to-CT time from 21.3 ± 16.7 min to 19.8 ± 16.7 min. This method increased the percentage of people achieving the goal of having a CT in less than 20 min from ED admission from 55.3% to 63.2% [24]. Similarly, Heiberger et al. (2019) established an approach team led by a nurse aimed at suspected stroke victims that helped reduce the average door-to-CT time from 17.08 min to 8.52 min. They concluded that the nursing team and the medical team’s synergistic collaboration led to an improvement in stroke approach time [25]. Madhok et al. (2019) implemented a protocol that involved a specialised team approaching the victims as they left the ambulance and referring them directly to CT. This method helped them reduce the average door-to-CT time from 19 min to 9 min [20].

In Portugal, the subject under investigation has yet to be studied. We came across an article by Pereira et al. (2017) that partially addressed the issue. However, the study was conducted between 2010 and 2012, and the triage system and guidelines differed from the current ones. As a result, it is not possible to compare their results with the present study. Nonetheless, it is worth noting that the survey highlighted the low percentage of VVAVC activation at the time. It emphasised the importance of quicker triage, assessment, and guidance of stroke victims in the acute phase to optimise diagnosis and treatment within the therapeutic window [26].

Regarding the second question, which investigates whether sociodemographic variables such as age and gender, as well as the clinical variable of previous mRS, have an impact on the activation of VVAVC, it was discovered that they were not significant factors in terms of the activation or non-activation of VVAVC. As no studies supported this question, future research should be conducted to determine which variables may be responsible for the percentage of non-activation of the VVAVC.

## 5. Implications for the Future and for International Audiences

This study underscores the importance of conducting further research to understand the factors that influence the activation of VVAVC during triage by nurses and how early VVAVC activation in the pre-hospital setting impacts outcomes after hospital admission. Adhering to the time-sensitive criteria outlined by the DGS, there are opportunities to enhance several steps in approaching a suspected stroke victim. It is essential to address the integration of workflows between emergency medical departments and hospital-based care. Our objective is to elevate the quality of care and improve health outcomes by developing and implementing a VVAVC activation protocol, as well as providing training to the multidisciplinary team caring for stroke victims in the ED. We also intend to conduct a comparative study to assess the benefits of these strategies once they are implemented. Additionally, we are working to establish a quality improvement feedback loop to enable continuous monitoring and iterative improvements to stroke care protocols.

The findings of this study have significant implications for international stroke care practises. The results highlight the critical need for timely activation of stroke protocols and suggest that efficient triage and immediate activation of VVAVC can substantially reduce door-to-CT time, potentially improving outcomes for stroke patients. Further research should explore the barriers to protocol activation and develop strategies to overcome them, ensuring that stroke victims receive prompt and effective care worldwide.

## 6. Limitations of the Study

Firstly, there is a lack of recent research on stroke in Portugal, and current statistical data on the prevalence and incidence of stroke are insufficient. This limitation hinders the comparison of data, but it also makes the study pioneering, relevant, and a way to improve care for stroke patients.

Additionally, the absence of data on pre-hospital VVAVC activations made it impossible to determine the impact of pre-hospital activation on door-to-CT time. This highlights the importance of establishing a centralised stroke database in Portugal to track stroke care from symptom onset to long-term follow-up.

## 7. Conclusions

This study highlights the pressing need to develop and implement a well-defined protocol with clear criteria for activating VVAVC at an institutional level. It is also essential to establish an in-hospital workflow for suspected stroke victims, which involves a multidisciplinary team with specialised training and updated knowledge of the latest ESO guidelines. This study’s findings advocate for a systematic approach to stroke management that speeds up the process and tailors care to each individual’s needs, ultimately aiming to improve stroke-related outcomes.

## Figures and Tables

**Table 1:** Sociodemographic characterisation of cases identified by year (2021–2022).

Year	2021		2022		Total		Residuals		X^2^	*p*
Variables	n 115	% 50.9	n 111	% 49.1	n 226	% 100	2021	2022		
Age									2.774	0.428
≤72 years	33	28.7	23	20.7	56	24.8	1.4	−1.4		
73–82 years	32	27.8	29	26.1	61	27.0	0.3	−0.3		
83–89 years	26	22.6	33	29.7	59	26.1	−1.2	1.2		
>89 years	24	20.9	26	23.4	50	22.1	−0.5	0.5		
Gender									0.013	0.909
Male	62	53.9	59	53.2	121	53.5	0.1	−0.1		
Female	53	46.1	52	46.8	105	46.5	−0.1	0.1		
Total	115	100	111	100	226	100				

n—Number; %—Percentage.

**Table 2:** Clinical characterisation (pre-admission) of identified cases by year (2021–2022).

Year	2021		2022		Total		Residuals		X^2^	*p*
Variables	n 115	% 50.9	n 111	% 49.1	n 226	% 100	2021	2022		
Previous mRS									3.992	0.551
No symptoms	81	70.4	78	70.3	159	70.4	0.0	0.0		
No significant disability	4	3.5	9	8.1	13	5.8	−1.5	1.5		
Slight disability	3	2.6	5	4.5	8	3.5	−0.8	0.8		
Moderate disability	14	12.2	11	9.9	25	11.1	0.5	−0.5		
Moderate severe disability	11	9.6	7	6.3	18	8.0	0.9	−0.9		
Severe disability	2	1.7	1	0.9	3	1.3	0.6	−0.6		
Origin									3.607	0.165
Outside	10	8.7	17	15.3	27	11.9	−1.5	1.5		
CODU	95	82.6	89	80.2	184	81.4	0.5	−0.5		
Health Centre	10	8.7	5	4.5	15	6.6	1.3	−1.3		
Total	115	100	111	100	226	100				

n—Number; %—Percentage; mRS—Modified Rankin Scale; CODU—Centre of Portuguese Urgent Patients Orientation.

**Table 3:** Clinical characterisation (post-admission) of identified cases by year (2021–2022).

Year	2021		2022		Total		Residuals		X^2^	*p*
Variables	n 115	% 50.9	n 111	% 49.1	n 226	% 100	2021	2022		
Shift									4.345	0.114
Night (0–8 h)	5	4.3	12	10.8	17	7.5	−1.8	1.8		
Morning (8–16 h)	65	56.5	65	58.6	130	57.5	−0.3	0.3		
Afternoon (16–24 h)	45	39.1	34	30.6	79	35.0	1.3	−1.3		
Moment of VVAVC activation									8.317	0.016 *
Triage	32	27.8	51	45.9	83	36.7	−2.8	2.8		
Post-triage	16	13.9	14	12.6	30	13.3	0.3	−0.3		
Non-activation	67	58.3	46	41.4	113	50.0	2.5	−2.5		
Total	115	100	111	100	226	100				

* *p* < 0.05—statistically significant; n—Number; %—Percentage; VVAVC—“*Via Verde Acidente Vascular Cerebral*”.

**Table 4:** Statistics on evaluated times according to year (2021–2022).

Times		2021		2022		2021–2022	
		Mean	Sd	Mean	Sd	Mean	Sd
Admission to triage		0:08	0:05	0:09	0:05	0:08	0:05
Door-to-CT	VVAVC Triage	0:28	0:12	0:39	0:19	0:35	0:18
	VVAVC Post-Triage	0:32	0:14	0:44	0:34	0:38	0:26
	VVAVC Non-activated	1:09	0:51	0:56	0:34	1:04	0:45
	TOTAL	0:52	0:44	0:47	0:29	0:50	0:37
Admission to VVAVC activation		0:06	0:15	0:05	0:17	0:05	0:16

Sd—Standard Deviation; VVAVC—“Via Verde Acidente Vascular Cerebral”; Door-to-CT—Door-to-Computerised-Tomography.

**Table 5:** Kruskal–Wallis test relating the moment of VVAVC activation with the Door-to-CT time.

	Door-to-CT Time			Test
	Average Ranking	Mean ^1^	Sd	
Moment of activation Triage Post-triage Non-activated	86.57 89.02 139.78	0:35 0:38 1:04	0:18 0:26 0:45	Kruskal–Wallis
(*p*)	0.000 *			

* *p* < 0.05—statistically significant; Door-to-CT—Door-to-Computerised-Tomography; Sd—Standard Deviation; ^1^ 2021–2022.

**Table 6:** Variation in the moment of activation of VVAVC according to age, gender, and previous mRS.

Moment of VVAVC Activation	Triage (T)		Post-Triage (P-T)		Non-Activation (NA)		Residuals			X^2^	*p*
Variables ^1^	n 83	% 36.7	n 30	% 13.3	n 113	% 50.0	T	P-T	NA		
Age										6.247	0.396
≤72 years	19	22.9	12	40.0	25	22.1	−0.5	2.1	−0.9		
73–82 years	20	24.1	8	26.7	33	29.2	−0.7	0.0	0.7		
83–89 years	26	31.3	5	16.7	28	24.8	1.4	−1.3	−0.5		
>89 years	18	21.7	5	16.7	27	23.9	−0.1	−0.8	0.6		
Gender										2.834	0.242
Male	48	57.8	12	40.0	61	54.0	1.0	−1.6	0.1		
Female	35	42.2	18	60.0	52	46.0	−1.0	1.6	−0.1		
Previous mRS										14.539	0.150
No symptoms	60	72.3	26	86.7	73	64.6	0.5	2.1	−1.9		
No significant disability	6	7.2	1	3.3	6	5.3	0.7	−0.6	−0.3		
Slight disability	5	6.0	0	0.0	3	2.7	1.5	−1.1	−0.7		
Moderate disability	5	6.0	3	10.0	17	15.0	−1.8	−0.2	1.9		
Moderate severe disability	7	8.4	0	0.0	11	9.7	0.2	−1.7	1.0		
Severe disability	0	0.0	0	0.0	3	2.7	−1.3	−0.7	1.7		

n—Number; %—Percentage; VVAVC—“*Via Verde Acidente Vascular Cerebral*”; mRS—Modified Rakin Scale; ^1^ 2021–2022.

## Data Availability

The data generated and analysed during the current study are not publicly available due to the need to protect patient privacy and comply with ethical guidelines. However, the main statistical data are summarised throughout the paper. Requests for access to data for research purposes can be considered upon reasonable request.

## Public Involvement Statement

No public involvement in any aspect of this research.

## Guidelines and Standards Statement

This manuscript was drafted against the STROBE STrengthening the Reporting of OBservational studies in Epidemiology research.

## Use of Artificial Intelligence

This manuscript utilised artificial intelligence (AI) tools and methodologies to enhance the research and writing processes. Specifically, AI was employed in the following areas: 1. ata Analysis: AI algorithms were used to analyse the large dataset, providing deeper insights and identifying patterns that manual analysis might have overlooked. Tools such as IBM SPSS^®^ and Python libraries were instrumental in performing statistical analyses and visualisations. 2. Literature Review: AI-powered tools facilitated an extensive and thorough literature review by quickly identifying relevant studies and summarising key findings, ensuring that the manuscript was informed by the latest research. 3. Writing Assistance: AI-based writing assistants helped in drafting and editing the manuscript, improving the clarity, coherence, and grammatical accuracy of the text. These tools also helped in adhering to the specific formatting and style guidelines required by the journal. 4. Translation and Multilingual Support: AI translation services were employed to ensure that the manuscript could be accurately translated into other languages if required, making the research accessible to a broader audience. The incorporation of AI technologies has significantly contributed to the efficiency and quality of this research, demonstrating the potential of AI in advancing scientific inquiry and academic publishing.
